# Pressure Time Dose as a Representation of Intracranial Pressure Burden and Its Dependency on Intracranial Pressure Waveform Morphology at Different Time Intervals

**DOI:** 10.3390/s23198051

**Published:** 2023-09-24

**Authors:** Anna-Li Schönenberg-Tu, Dirk Cysarz, Benjamin Petzold, Carl Benjamin Blümel, Christa Raak, Oliver Fricke, Friedrich Edelhäuser, Wolfram Scharbrodt

**Affiliations:** 1 Chair of Integrative Neuro-Medicine, Gemeinschaftskrankenhaus Herdecke, 58313 Herdecke, Germany; 2 Institute of Integrative Medicine, Witten/Herdecke University, 58313 Herdecke, Germany; 3 Integrated Curriculum for Anthroposophic Medicine, Witten/Herdecke University, 58313 Herdecke, Germany; 4 Faculty of Health, Department of Human Medicine, Witten/Herdecke University, 58455 Witten, Germany; 5 Department of Child and Adolescent Psychiatry and Psychotherapy, Klinikum Stuttgart, 70174 Stuttgart, Germany

**Keywords:** intracranial pressure, waveform analysis, algorithm design, ICP burden, pressure time dose, area under the curve, time intervals

## Abstract

Intracranial pressure (ICP) burden or pressure time dose (PTD) is a valuable clinical indicator for pending intracranial hypertension, mostly based on threshold exceedance. Pulse frequency and waveform morphology (WFM) of the ICP signal contribute to PTD. The temporal resolution of the ICP signal has a great influence on PTD calculation but has not been systematically studied yet. Hence, the temporal resolution of the ICP signal on PTD calculation is investigated. We retrospectively analysed continuous 48 h ICP recordings with high temporal resolution obtained from 94 patients at the intensive care unit who underwent neurosurgery due to an intracranial haemorrhage and received an intracranial pressure probe (43 females, median age: 72 years, range: 23 to 88 years). The cumulative area under the curve above the threshold of 20 mmHg was compared for different temporal resolutions of the ICP signal (beat-to-beat, 1 s, 300 s, 1800 s, 3600 s). Events with prolonged ICP elevation were compared to those with few isolated threshold exceedances. PTD increased for lower temporal resolutions independent of WFM and frequency of threshold exceedance. PTD_beat-to-beat_ best reflected the impact of frequency of threshold exceedance and WFM. Events that could be distinguished in PTD_beat-to-beat_ became magnified more than 7-fold in PTD_1s_ and more than 104 times in PTD_1h_, indicating an overestimation of PTD. PTD calculation should be standardised, and beat-by-beat PTD could serve as an easy-to-grasp indicator for the impact of frequency and WFM of ICP elevations on ICP burden.

## 1. Introduction

‘ICP burden’ or ‘pressure time dose’ (PTD) of intracranial pressure (ICP) includes both the magnitude and the duration of ICP elevations and has been shown to be a suitable indicator for the impact of ICP elevation on clinical outcome (e.g., occurrence of secondary brain insults) [1]. The original method to calculate the area under the curve (AUC) of vital parameter signals via trapezoidal interpolation was first employed for estimating hypotension load in ambulatory blood pressure [1] and fever burden load [2]. PTD has been shown to be correlated with poor outcome as quantified by mortality rate [3], Glasgow Outcome Scale (GOS) [4], length of stay in the intensive care unit (ICU) [5] and qualitatively worse neurological outcome [6], and could therefore guide clinical decision making with regard to more aggressive treatments [7].

ICP-based care to date has mainly been based on thresholds that have been found to be potentially harmful to the brain, at least in the long term. Due to the complexity and irregularity of the ICP signal, there is a constant search for predictive properties of the ICP curve that can be represented in indices of ICP, of which PTD is one. The concept of PTD aims to combine threshold exceedance with duration to achieve a notion of total dose. Lower exceedances for a longer duration of time can thus accumulate to a higher PTD than higher exceedances for a shorter duration of time.

Most methods to evaluate PTD focus on the duration and intensity that ICP exceeds a certain threshold [8,9,10] without considering other properties of the ICP signal such as the waveform morphology. Furthermore, with 20 mmHg as the most common cut-off [3], the weak predictive potential of PTD for clinical outcome was attributed to the rather low temporal resolution (60, 30 or 15 min in many ICU documentation systems). Only calculations with a relatively high temporal resolution (sampling interval: 1 min) were correlated with GOS [9] and allowed for a more precise assessment and prediction of the clinical outcome [11]. However, many data documentation systems in the ICU use a rather low temporal resolution of the ICP signal for PTD calculations and, hence, the clinical utility of PTD, although widely used, may be questionable.

Calculations of PTD with low temporal resolution of the ICP are very limited in showing the impact of shorter spikes in the ICP signal. Furthermore, the ICP waveform morphology has not been considered in calculations, despite the fact that it is known to change during cerebral stress. Changes in waveform morphology during cerebral stress may involve a change in peak occurrences within a single ICP wave. The second peak may become higher than the first peak in each ICP wave, indicating a reversal of the peak height [12], changes in macro patterns with increasing slow waves and plateau building [13] and changes in pulsatile components such as an increase in amplitude and steepness [12,14]. Such changes are known to be indicators of diminishing cerebral compliance and impending threats of structural brain damage.

It can be assumed that a single elevated ICP wave above a certain threshold or a lasting ICP elevation over a certain period of time can be detrimental to the various structures within the skull, including the brain tissue. Hence, the frequency and shape of such elevations may be relevant in determining how damaging these ICP elevations are.

**Figure 1:**
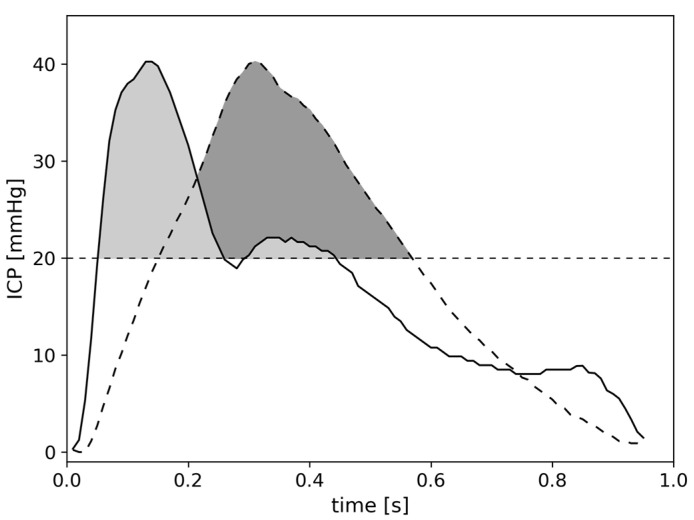
Example of the AUC for a narrow and broad ICP wave. The AUC above the threshold of 20 mmHg (straight dashed line) is obviously influenced by the shape of the ICP wave. The PTD of the narrow wave (solid line) is 4.98 mmHg·s (cf. light grey area) whereas the PTD of the broad wave (dashed line) is 6.41 mmHg·s (cf. dark grey area), with both waves reaching the same peak value. If the PTD is calculated with a temporal resolution of, e.g., 1 s and according to the maximal ICP value multiplied by its duration, both waves would result in the same PTD: 20.24 mmHg·s.

The predictive potential of the ICP waveform has been investigated as a means of forecasting impending intracranial hypertension [15], but the relationship between waveform morphology and ICP burden has not been thoroughly analysed yet. It can be postulated that the overall burden of ICP elevation can be more accurately quantified by the cumulative AUC of the individual ICP waves than by summing up the times that ICP exceeds a given threshold. Beat-to-beat calculations of cumulative PTD would also allow one to include the impact of the shape and frequency of ICP elevations. Figure 1 illustrates how PTD calculated by the AUC reflects the entire waveform of the ICP signal in contrast to the calculation of the PTD according to the maximal ICP value times its duration. In the example, the latter calculation overestimates the PTD clearly. With a steeper, narrower shape of the ICP wave, the AUC representing PTD still remains comparatively low, while the broader shape of the ICP wave increases the AUC and therefore PTD.

Our objective is to show that the temporal resolution of the ICP signal and the waveform morphology contribute to the overall dose of ICP, whereas the simple calculation according to the maximal ICP value times its sampling interval overestimates the PTD. The cumulative area under the curve of the beat-to-beat ICP signal would then allow greater accuracy in the early detection of an impending critical ICP elevation and could potentially be used for clinical decision making. PTD can thus give an easily accessible clinical indicator of the effects of waveform morphology, which are otherwise difficult to detect. This can also be helpful for guiding clinical decision making in sub-level ICP increases.

## 2. Materials and Methods

### 2.1. Dataset and Study Population

In this study, we analysed datasets obtained from 94 patients (43 females, median age: 72 years, age range: 23 to 93 years). They had been admitted between January 2017 and March 2021 with non-traumatic intracranial haemorrhage, severe enough that their clinical management required ICP monitoring at the ICU and justified invasive probe according to the usual standards [16,17]. All patients needed to have at least one interval of intracranial hypertension within the appraisal interval. The exclusion criteria were age under 18 years for judicial reasons, accompanying intracranial infections and craniectomy having taken place either during or before the monitoring period or a continuous recording period of less than 48 h. Informed written consent was obtained from all of the patients included in the study either by themselves or by their legal representatives. Data were anonymised before further analysis. The study was approved by the ethical committee board of the University of Witten/Herdecke (178/2015).

The monitoring data of all vital parameters of all patients in our ICU that underwent ICP monitoring were recorded from the patient monitor (Dräger Infinity Delta XL, Lübeck, Germany) using an exchange server (Dräger Infinite Central Station, Lübeck, Germany) that permitted the recording of the data on an external server for subsequent analysis. The recording of all of the vital parameters was terminated after the patients were no longer in need of ICP monitoring, usually either because they could be referred to another department or because they died due to the severity of the illness. The ICP signal was obtained via an invasive intraparenchymal pressure probe (Raumedic Neurovent-P, Helmbrechts, Germany) in the left or right frontal scull. The other recorded parameters comprised electrocardiogram (ECG), pulse oximetry and invasively registered arterial blood pressure (ABP) at the height of the atria (CODAN pvb, Lehnsahn, Germany), including mean arterial pressure (MAP), core body temperature and details of ventilation parameters such as positive end-expiratory pressure (PEEP) and peak ventilating pressure. ECG was recorded with a sampling rate of 200 Hz, and ICP and ABP were recorded with a sampling rate of 100 Hz. The signals were recorded for 48 h. Clear artefacts in the ECG were manually excluded from further analysis. The algorithms to reliably identify and record each ICP wave and other corresponding vital parameters such as ECG, ABP, temperature and modes of ventilation have continuously been developed and refined. The local minimum in the ICP signal is defined as the start of a new ICP wave and the end of the preceding ICP wave. It was determined in a time window of 200 ms after the R-peak appeared in the ECG. We recently compared our algorithm to identify ICP waves to the modified algorithm developed by Scholkmann et al. [18,19] with respect to artefact resistance and start-point identification accuracy, and showed that it provides higher sensitivity to correctly identify ICP waves and lower overall error rates (<0.5%) [20]. The low error rates permitted the use of the start-points of the ICP waves without further correction. In this study, <0.1% of all ICP waves were discarded because the ECG was contaminated by artefacts and, hence, the R-peak that precedes the ICP wave could not be identified unambiguously. Consequently, the corresponding ICP waves could not be identified and they were discarded.

### 2.2. Calculation of ICP Burden as Pressure Time Dose

We calculated and accumulated the area under the curve (AUC) for the continuous ICP signal considering each ICP wave. This calculation was deemed as the most accurate calculation of real-time, overall ICP burden. We assumed that the beat-to-beat resolution as the base for calculating ICP burden should be more resistant to artefacts, since the algorithm excludes any part of the ICP signal without a proper ICP waveform. We therefore did not design any extra features to filter out plateaus like those which occur with an ICP probe being disconnected or recalibrated, which usually lie in the range of 200 mmHg to 600 mmHg. To consider only clinically relevant elevated ICP and the potentially damaging effects of ICP burden, the AUC was calculated for the part of the ICP time series that exceeded a threshold of 20 mmHg, using Simpson’s rule (cf. Figure 1). This calculation is the most realistic assessment of ICP burden and will be used as the reference throughout. The AUC reflecting the cumulative pressure time dose (PTD in mmHg·s) [9] was denoted as PTD_beat-to-beat_.

Since we specifically wanted to investigate the relevance of sampling intervals for obtaining ICP values to calculate PTD, we compared PTD_beat-to-beat_ to ICP readings of larger temporal resolution. We simulated manual recordings of the ICP curve as usually practiced in nursing flow sheets by recording end-of-hour values. The numerical value of ICP was taken at pre-defined time intervals, with the largest being one hour and the shortest being one second. To show the progression, we used time intervals of 1 h, 30 min, 5 min, 1 s and beat-by-beat which were denoted and indexed as follows: PTD_1h_, PTD_30min_, PTD_5min_, PTD_1s_ and PTD_beat-to-beat_, respectively.

**Figure 2:**
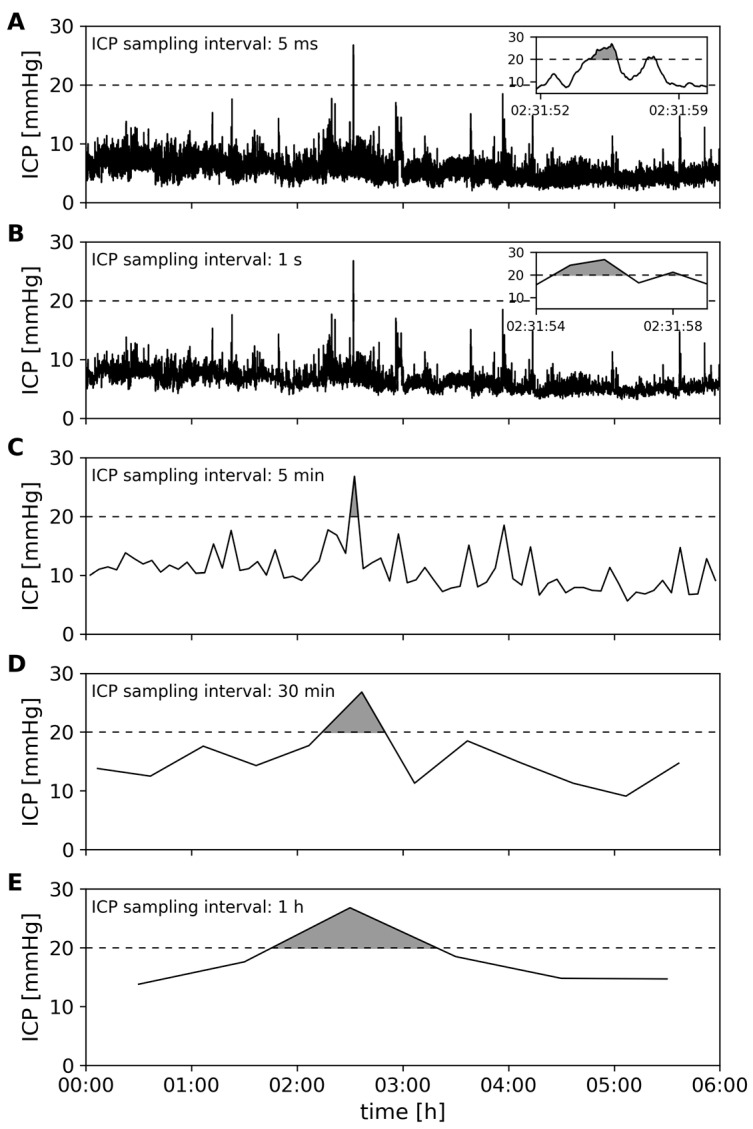
Examples of AUC calculation for the entire beat-to-beat ICP signal at sampling rate of 100 Hz ((**A**): inset diagram shows the only occurrence of ICP > 20 mmHg relevant for the PTD calculation), maximal ICP at 1 s time interval ((**B**): inset diagram shows ICP > 20 mmHg), 5 min time interval (**C**), 30 min time interval (**D**) and 1 h interval (**E**). The AUC at the time interval of 1 s to 1 h (diagrams (**B**–**E**)) is considerably larger compared to the AUC from the entire ICP signal (diagram (**A**)).

Figure 2 illustrates how ICP burden derived from PTD_beat-to-beat_ translates into indices derived from recordings with larger temporal resolution. As can be seen, the larger the time interval, the larger the part of the ICP signal that is used for PTD calculation, potentially increasing its total amount. PTD_beat-to-beat_, on the other hand, has a much higher temporal resolution than the other indices of PTD, and the AUC would therefore show a higher variance for the individual calculations.

### 2.3. Statistical Analysis

The AUC of the ICP values at different time intervals does not show a Gaussian distribution because the elevation of the ICP signal above 20 mmHg depends on cerebral autoregulation that does not lead to Gaussian properties of a distribution of ICP values.

We thus used non-parametric statistical procedures. The distributions of PTD_1h_, PTD_30min_, PTD_5min_, PTD_1s_ and PTD_beat-to-beat_ were described using the median, 25% and 75% percentiles (inter quartile range, IQR). Variations between the distributions of PTD at different time intervals were calculated using the paired non-parametric analysis of variance (Friedman test). In case of significant variations, pairwise comparisons between the different time intervals were carried out post hoc, including adjustments for multiple comparisons according to Conover [21]. *p* < 0.05 was considered to be statistically significant.

## 3. Results

The median and interquartile range of PTD_beat-to-beat_, PTD_1s_, PTD_5min_, PTD_30min_ and PTD_1h_ in mmHg s are listed in Table 1. Generally, the PTD values increased with larger recording time intervals. The median ICP burden was the lowest for PTD_beat-to-beat_ (6457 mmHg·s) and the highest for PTD_1h_ (2,386,572 mmHg·s). The Friedman test indicated clear differences between the PTD values of the different time intervals (*p* < 0.001). The post hoc comparison between the PTD values of each temporal resolution showed significant differences between all of the temporal resolutions (*p* < 0.001).

The PTD increased with a larger temporal resolution. The PTD derived at larger time intervals was never smaller than the ICP burden calculated at a higher temporal resolution, i.e., smaller time intervals. All of the PTD values increased with a lower temporal resolution (i.e., larger time intervals) as well as the quotient, which represents the relationship from one time-scale to the next. Isolated spikes of intracranial hypertension that have little impact on PTD_beat-to-beat_ became magnified up to 7-fold in PTD_1s_ and up to 2000-fold in PTD_1h_. The proportion of distortion when moving on from PTD_beat-to-beat_ to PTD_1s_ and further up to larger time intervals is irregular. A pattern or model that would allow for the backward deduction of PTD_beat-to-beat_ from larger temporal resolution indices is not discernible. A backward deduction of ICP burden from lower to higher temporal resolution indices is therefore not possible, and so taking lower temporal resolution values will not allow for an accurate estimation of the “true” PTD.

The relative increase in PTD with decreasing temporal resolution (PTD1s, PTD5min, PTD30min and PTD1h) in relation to the reference PTD_beat-to-beat_ is shown in Table 2. The median increases in PTD were 2.4 (PTD_1s_/PTD_beat-to-beat_), 28.8 (PTD_5min_/PTD_beat-to-beat_), 129.7 (PTD_30min_/PTD_beat-to-beat_) and 243.6 (PTD_1h_/PTD_beat-to-beat_). The Friedman test again indicated clear differences between each time interval (*p* < 0.001). The post hoc comparison of the relative increase at the different time intervals showed that most of the comparisons yielded *p*-values < 0.001.

**Figure 3:**
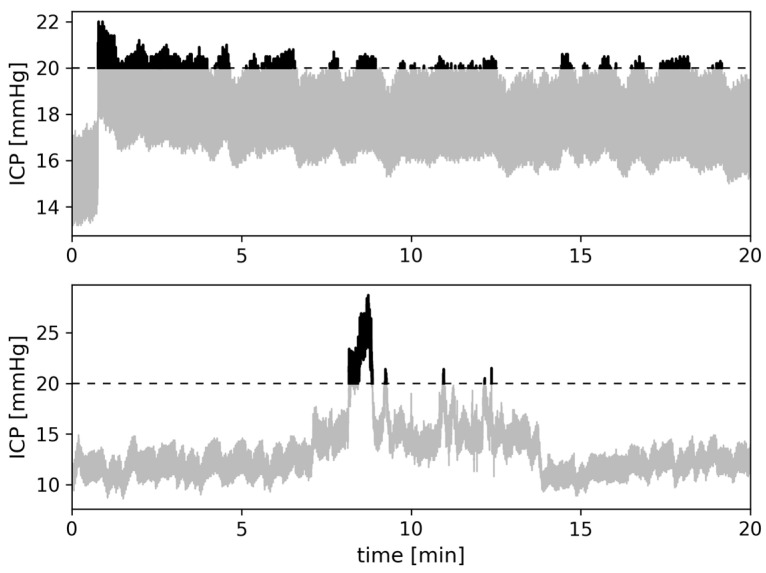
Examples of ICP time series obtained from two patients. In the upper diagram, ICP frequently exceeds the threshold of 20 mmHg (dashed line) as seen via the frequent short peaks (accumulated PTD_beat-to-beat_: 12 mmHg·s). In the lower diagram, ICP exceeds 20 mmHg less frequently but the peak ICP is far higher and broader than in the upper diagram (accumulated PTD_beat-to-beat_: 130 mmHg·s).

Figure 3 illustrates how a single spike of intracranial hypertension within a 20 min recording interval if taken at face-value can be much further apart from “true” PTD (i.e., PTD_beat-to-beat_) in one case than in another. A closer look at the shape and frequency of the intracranial hypertension episodes of two examples shows big differences: the signal in the upper diagram frequently exceeds the threshold of 20 mmHg, but these exceedances are of a short duration and, hence, do not add up to a very high PTD. The example in the lower diagram shows one longer-lasting exceedance of the threshold that contributes to a much higher overall PTD. A qualitative comparison of the ICP signals in both diagrams suggests that the PTD is higher in the ICP trace of the upper diagram than in the ICP trace of the lower diagram. However, calculating PTD_beat-to-beat_ reveals that the accumulated PTD in the upper diagram’s ICP signal is far lower than in the lower diagram’s ICP signal (12 mmHg·s vs. 130 mmHg·s). The PTD of the lower diagram even becomes magnified if the temporal resolution is lowered (cf. Figure 2).

## 4. Discussion

We have demonstrated that the total burden of ICP elevation can be more accurately quantified by the cumulative AUC of each ICP exceedance compared to the time the ICP exceeds a given threshold multiplied by the ICP maximum. Generally, the larger the time intervals of the ICP signal, i.e., the lower the temporal resolution, the higher the PTD, since even short events of intracranial hypertension may have an impact for at least the time order of the temporal resolution. As a result, the PTD quantified from low-temporal-resolution recordings may overestimate the “true” PTD considerably and would suggest an advancement of aggressive treatment too early [11].

At a lower temporal resolution, single elevated ICP values could theoretically be both overestimated and even omitted, depending on whether or not they happen to be in the frame in which the algorithm picks them up. However, the study shows that larger temporal resolution always results in overemphasis of singular ICP elevations in relation to ICP burden, and never in an underrepresentation.

Larger time intervals lead to simpler and less detailed time series of the ICP, and a backward deduction of signal values at larger timer intervals to signal values at shorter time intervals is not possible (cf. Figure 2). Consequently, ICP recordings using larger time intervals for sampling neither allow for the backward-tracking of single, short-term events that occur within the time interval nor do they allow for refinement in order to calculate a more accurate estimation of ICP burden. On the other hand, even relatively low ICP values can have a significant effect on PTD if the waveform morphology changes, i.e., the broadening of single waves or a change in the distribution of the peaks within a single ICP wave. The degree to which the ICP burden changes with larger temporal resolution is different depending on the pulse rate and underlying waveform morphology, such as the P2/P1 ratio. Hence, beat-by-beat calculation of the AUC best reflects the ICP burden.

Our findings are consistent with other studies in which the AUC of the ICP curve was shown to provide a good representation of the ‘dose’ of ICP elevation [6,10] and be correlated with the Glasgow Outcome Scale (GOS). Prior investigations using the trapezoid method on an ICP curve were based on manual recordings of end-of-hour values [6,8] or the relationship with elevated minute-by-minute recordings of ICP [8], and imply that it is not only the elevation of ICP over a certain threshold that is relevant to the clinical outcome but also the duration and frequency of the ICP increase. In previous studies, waveform morphology only showed weak predictive power for intracranial hypertension and clinical outcome [14], but was shown to be correlated with overall ICP burden, such as a change in the magnitude of ICP peaks [22] or a loss in complexity of the ICP waveform [23]. Hence, the general clinical usefulness of the calculation of ICP burden considering the entire ICP waveform morphology remains to be confirmed. Formerly, a weakness of high-temporal-resolution ICP recordings was their susceptibility to all kinds of artefacts. Temporal resolution has been addressed as a potentially primary cause for a lack of a distinct correlation, as reported by Hemphill et al.: “High frequency data acquisition may be necessary for more precise evaluation of secondary brain injury in neurocritical care” [11]. This issue could only be addressed appropriately in recent years with the development and employment of appropriate filters.

It is still not quite clear whether an unambiguous causality of the ICP waveform with cerebral compliance exists or whether they are both only correlated indicators of progressive brain damage. Nevertheless, there is evidence that waveform morphology can be useful in order to detect impaired autoregulation and structural brain damage to at least some degree [11]. Furthermore, there are distinct changes in ICP waveform morphology that have been described to coincide with structural brain damage [12,13,14]. However, these changes are very specific and can often only be recognised and assessed by clinicians familiar with neurocritical care—a specialty that is not always available 24/7 in most ICUs. It is therefore necessary to develop easily comprehensible markers for clinicians and nurses involved with but not specialised in neurocritical care, and PTD_beat-to-beat_ might be an appropriate candidate.

With real-time analysis of the entire ICP signal, it is possible to consider the impact of waveform morphology on PTD. PTD_beat-to-beat_ includes information regarding waveform morphology and, hence, allows one to grasp the impact of waveform morphology changes more intuitively. PTD_beat-to-beat_ will be included in future clinical studies and, in particular, the correlation between PTD_beat-to-beat_ and clinical outcome will be investigated. In addition, different thresholds for calculating PTD_beat-to-beat_ (e.g., 16 mmHg, 18 mmHg or 22 mmHg) could be used and investigated with respect to their clinical utility.

Our analysis shows that the beat-to-beat analysis of the AUC provides the most accurate calculation of PTD to date and should be considered for PTD assessment in other studies. If the PTD_beat-to-beat_ is of clinical value, it could be displayed as a default parameter on ICU monitors. Further clinical research should also clarify whether PTD_beat-tobeat_ is better correlated with clinical outcome than other commonly used PTD calculations with temporal resolutions of intensive care medical data documentation systems used in clinical practice. If so, then ICU data management systems should change their temporal resolution for PTD calculations accordingly. Considering the entire waveform morphology of ICP signals may be necessary for machine learning to identify unfavourable alterations of ICP waveform morphology.

## Figures and Tables

**Table 1:** Median and interquartile range (IQR) of PTD values (in mmHg·s) at different temporal resolutions.

	PTD_beat-to-beat_	PTD_1s_	PTD_5min_	PTD_30min_	PTD_1h_
Median	18,871	47,056	577,142	2,271,793	4,102,786
IQR (25%, 75%)	6457 74,102	15,041 160,240	338,378 1,181,806	1,251,590 3,830,288	2,386,572 7,206,278

*p_Friedman_* < 0.001; all pairwise comparisons: *p* < 0.001.

**Table 2:** Median and interquartile increase (IQR) in PTD at different temporal resolutions in relation to PTD_beat-to-beat_ (i.e., PTD_b2b_).

	PTD_1s_/PTD_b2b_	PTD_5min_/PTD_b2b_	PTD_30min_/PTD_b2b_	PTD_1h_/PTD_b2b_
Median	2.4	28.8	129.7	243.6
IQR (25%, 75%)	1.3 3.4	10.8 64.8	35.1 60.8	63.7 61.8

*p_Friedman_* < 0.001; all pairwise comparisons: *p* < 0.05.

## Data Availability

The data presented in this study are available upon reasonable request from the corresponding author.
